# A case of contrast-induced encephalopathy after thoracic endovascular aortic repair for a ruptured thoracic aortic aneurysm

**DOI:** 10.1186/s44215-026-00244-7

**Published:** 2026-03-05

**Authors:** Jumpei Tokutome, Yuichiro Kishimoto, Takeshi Onohara, Hiromu Horie, Tsuyoshi Sasami, Rikuto Nii, Naoki Sumi, Nozomi Kishimoto, Kenichi Morimoto, Junya Nakashima, Yasushi Yoshikawa

**Affiliations:** Department of Cardiovascular Surgery, Tottori University Faculty of Medicine, 36-1 Nishicho, Yonago, Tottori, 683-8504 Japan

**Keywords:** Contrast-induced encephalopathy, TEVAR, Contrast media, Cerebral edema, CKD

## Abstract

**Background:**

Contrast-Induced Encephalopathy (CIE) is a rare complication, most commonly reported after carotid or coronary interventions, possibly due to reporting bias.

**Case presentation:**

We present a case of CIE following two-debranching thoracic endovascular aortic repair (TEVAR). A 79-year-old man with hypertension, hyperlipidemia, stage 4 chronic kidney disease (CKD), and prior cerebral infarction underwent emergency 2-debranching TEVAR for a ruptured thoracic aortic aneurysm (TAA). A total of 200 mL of contrast medium was used pre- and intraoperatively. On postoperative day (POD) 1, the patient remained unconscious despite sedation cessation. Non-contrast brain Computed tomography (CT) showed left hemispheric cerebral edema and high-density areas suggestive of contrast extravasation, raising suspicion for CIE. Conservative management with hydration and supportive care led to gradual recovery, with imaging on POD9 showing near-complete resolution.

**Conclusions:**

Although rare, CIE should be considered in cases of delayed consciousness recovery after TEVAR, particularly in high-risk patients. Early diagnosis and conservative treatment are essential for good outcomes.

## Background

CIE is a rare neurological complication caused by transient disruption of the blood–brain barrier (BBB) due to iodinated contrast media [1]. There have been more reports of CIE following cerebral or coronary interventions [2–5], although it is unclear whether these procedures are inherently more likely to cause CIE. Reported incidence ranges from 0.06% to 1% [6–9]. Occurrences following thoracic endovascular aortic repair (TEVAR) are exceedingly uncommon. Here in, we report a case of CIE after TEVAR in a patient with multiple risk factors and prolonged recovery.

## Case presentation

A 79-year-old man with a history of hypertension, hyperlipidemia, and CKD stage 4 (creatinine 2.04 mg/dL, estimated glomerular filtration rate (eGFR) 25.38 mL/min/1.73 m^2^), as well as prior cerebral infarction, presented with a ruptured TAA. A preoperative contrast-enhanced CT (100 mL ioversol) confirmed rupture. Emergency two-debranching TEVAR was performed, including bypass creation using an 8-mm T-shaped Expanded polytetrafluoroethylene (ePTFE) graft (W. L. Gore & Associates) from the right axillary artery to the left carotid and axillary arteries. Intraoperative rupture-induced hypotension required urgent stent graft deployment (W. L. Gore & Associates), stabilizing vital signs. The total operation time was 222 min, using 100 mL of iomeprol (300 mg/mL).

**Fig. 1:**
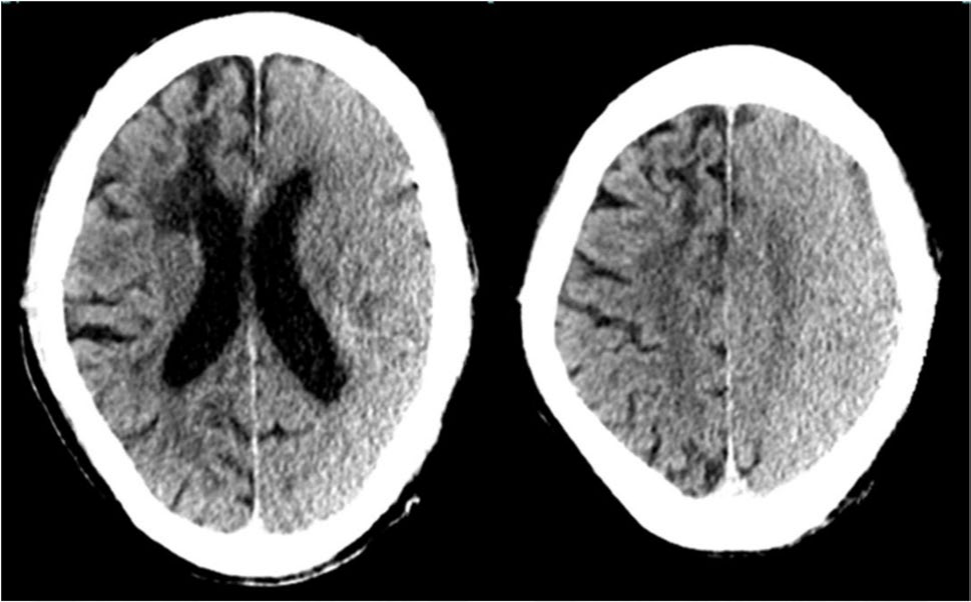
Non-contrast brain CT on POD1. CT showing sulcal effacement and high attenuation in the left hemisphere, indicating cerebral edema and suspected contrast leakage. CT: computed tomography. POD: postoperative day

**Fig. 2:**
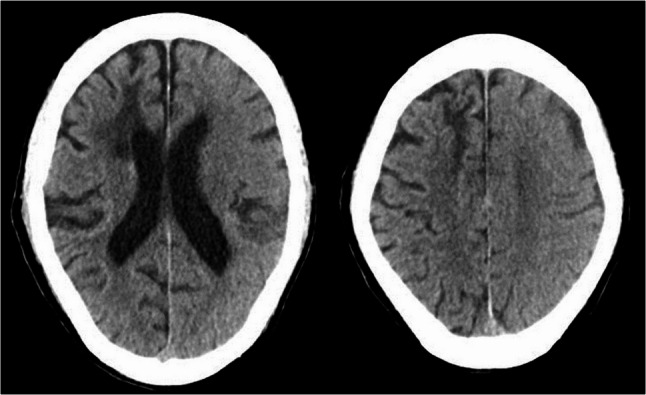
Non-contrast CT on POD2. CT showing partial resolution of edema and attenuation. CT: computed tomography. POD: postoperative day

**Fig. 3:**
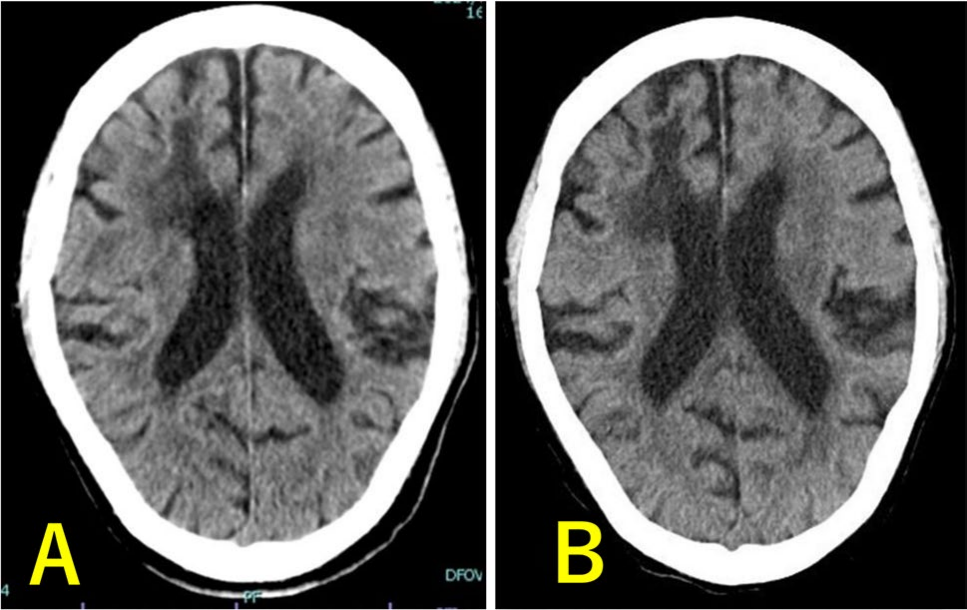
Preoperative CT and CT on POD9. **A** Preoperative CT. **B** POD9 CT. Showing resolution of edema and sulcal restoration. CT: computed tomography. POD: postoperative day

**Fig. 4:**
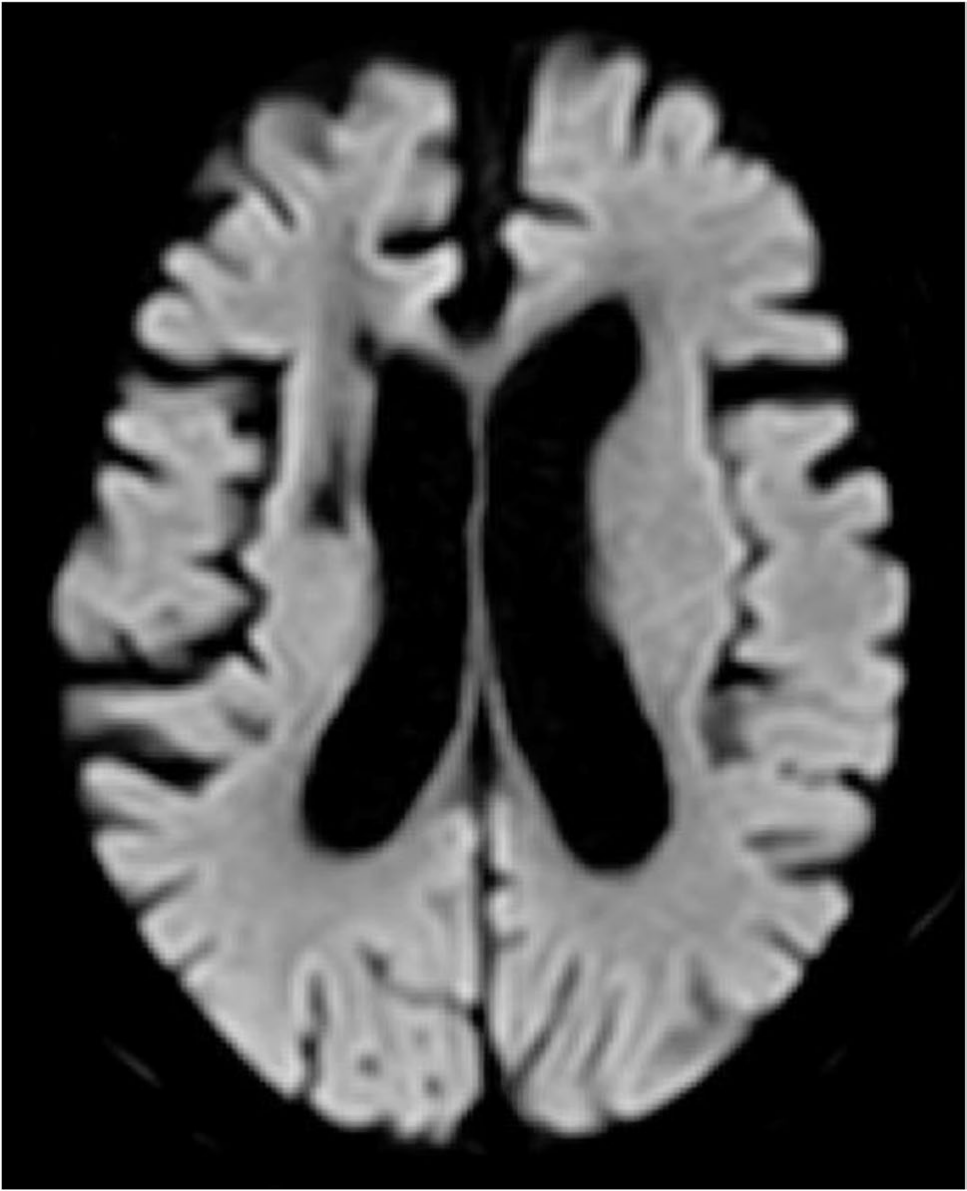
MRI in 2 months after surgery. MRI showing no new infarction; old infarct in right hemisphere only. MRI: magnetic resonance imaging

Postoperatively, the patient remained intubated and sedated with propofol in the Intensive Care Unit (ICU). Despite the cessation of sedation on postoperative day (POD) 1, the patient remained unresponsive. Brain CT revealed cerebral edema and high-density areas in the left hemisphere, suggestive of contrast extravasation (Fig. 1), without evidence of hemorrhage or infarction. His Glasgow Coma Scale (GCS) score was 8 (E3V1M4), indicating markedly impaired communication. Based on clinical and radiologic findings, CIE was strongly suspected. Conservative management including hydration and supportive care was initiated. MRI was deferred during the acute phase to minimize physiological burden. By POD2, CT showed reduced edema and sulcal effacement (Fig. 2). Neurological status gradually improved, and by POD4 the patient had recovered to a GCS score of 10 (E3V2M5). No new neurological deficits were observed. Follow-up CT on POD9 revealed near-complete resolution of edema (Fig. 3). His GCS had further improved to 14 (E4V4M6), and he was able to follow commands and engage in conversation. At two months, MRI showed no new infarction, only old ischemic changes (Fig. 4). The same MRI also demonstrated occlusion of the right anterior cerebral artery and mild stenosis of the right middle cerebral artery.

## Discussion and conclusion

This case illustrates a rare instance of CIE following TEVAR, a procedure not typically associated with cerebral complications. The patient presented multiple known risk factors for CIE: advanced CKD, prior stroke, hypertension, and intraoperative hypotension [3–5, 10, 11]. While typical CIE symptoms resolve within 24–48 h [10], our patient exhibited a delayed recovery over 60 h, despite cessation of sedation, indicating a prolonged course.

CIE is believed to result from BBB disruption and direct neurotoxicity of contrast media [1]. Findings typically include cortical/subarachnoid high attenuation and cerebral edema on CT [12].

It has been reported that CIE may present as either unilateral or bilateral involvement depending on the distribution of contrast flow. In previous literature, repeated injection of contrast into the internal carotid artery circulation tends to produce unilateral cortical involvement, whereas contrast administration predominantly into the posterior circulation is more likely to result in bilateral occipital abnormalities [3, 4, 6].

The vascular findings identified on postoperative MRI suggest that right-sided cerebral perfusion was chronically reduced. In combination with the left-sided coil embolization performed during TEVAR, these anatomical and procedural factors may have resulted in preferential contrast flow into the left cerebral circulation. This mechanism likely contributed to the extensive unilateral hemispheric involvement observed in this case. Despite dramatic initial findings, most cases show rapid radiologic and clinical improvement [1–3, 5, 6, 10, 12]. In our case, early follow-up imaging confirmed this trend. While contrast volume is implicated in CIE pathogenesis, reports exist of CIE even with lower doses [5]. The 200 mL used in this case (100 mL pre-op, 100 mL intra-op) was only slightly above the median reported TEVAR dose of 104 mL (IQR 69–168 mL) [13], underscoring that volume alone may not be the sole determinant.

MRI may support diagnosis but often yields non-specific or normal results [2–5]. In this case, follow-up MRI revealed no acute lesions. Importantly, CIE must be distinguished from stroke, especially when patients have delayed awakening post-TEVAR. On initial CT, cortical and subarachnoid high attenuation was observed, which is characteristic of CIE and not typical of acute ischemic stroke. No territorial hypodensity or loss of gray–white differentiation suggestive of infarction was identified. Furthermore, follow-up MRI revealed no acute ischemic lesions. These findings helped distinguish CIE from stroke as the cause of the patient’s delayed awakening. Given the risk of CIE even with standard contrast doses in at-risk patients, strategies such as minimizing contrast volume, optimizing renal function, and avoiding hypotension are essential.

Reports of CIE after TEVAR are extremely limited, and to our knowledge, Vallabhaneni et al. first reported a case of CIE following TEVAR [14]. Although CIE occurring after TEVAR is extremely rare, this case provides several important novel insights into the mechanisms and clinical characteristics of contrast-induced neurotoxicity. First, the striking unilateral distribution of contrast extravasation can be explained by a combination of hemodynamic and anatomical factors. During coil embolization, contrast was administered predominantly to the left side, and chronic right-sided hypoperfusion due to pre-existing cerebral arterial disease, including right ACA occlusion and mild right MCA stenosis, was also present. The interplay of these factors likely created a left-dominant contrast load, ultimately resulting in the markedly asymmetrical presentation of CIE observed in this case. To our knowledge, this mechanism has not been clearly described in previous reports.

Second, extensive unilateral cortical and subcortical contrast extravasation was documented across multiple time points using serial CT imaging, allowing visualization of the entire continuum from onset to near-complete resolution. Such detailed temporal imaging evolution has rarely been captured in the existing literature and provides valuable radiologic insight into CIE dynamics.

Third, this case illustrates how multiple predisposing factors, including advanced CKD, a prior stroke, and intraoperative hypotension, may act together to prolong neurological recovery despite eventual complete resolution. This underscores the importance of individualized risk assessment when evaluating delayed awakening after procedures involving iodinated contrast. Taken together, these features distinguish this case from previous reports and provide novel mechanistic, radiologic, and clinical insights into the spectrum of CIE following TEVAR.

While prognosis is generally favorable, delayed awakening warrants consideration of CIE alongside stroke. Early diagnosis and conservative management are key to successful outcomes. Clinicians should maintain a high index of suspicion for CIE particularly in patients undergoing TEVAR with predisposing factors such as CKD or prior stroke.

## Data Availability

The datasets used and/or analyzed during the current study are available from the corresponding author on reasonable request.

## Abbreviations

CIE: Contrast-Induced Encephalopathy
TEVAR: Thoracic endovascular aortic repair
CKD: Chronic kidney disease
TAA: Thoracic aortic aneurysm
POD: Postoperative day
CT: Computed tomography
BBB: Blood-brain barrier
eGFR: Estimated glomerular filtration rate
ePTFE: Expanded polytetrafuoroethylene
ICU: Intensive care unit
GCS: Glasgow Coma Scale
